# Scientific synergy between prophylactic and therapeutic HIV vaccines—prevention and cure

**DOI:** 10.1038/s41541-026-01538-1

**Published:** 2026-07-27

**Authors:** Milton Maciel, Lindsey R. Baden, Katharine J. Bar, Thomas Calder, Angela Malaspina, Maurine D. Miner, Stuart Z. Shapiro, M. Patricia D’Souza

**Affiliations:** 1 Division of AIDS, National Institute of Allergy and Infectious Diseases, National Institutes of Health, Rockville, MD USA; 2 Brigham and Women’s Hospital and Harvard Medical School, Boston, MA USA; 3 Perelman School of Medicine, University of Pennsylvania, Philadelphia, PA USA; 4 Fred Hutchtinson Cancer Center, Seattle, WA USA

**Keywords:** Diseases, Health care, Immunology, Medical research, Microbiology

## Abstract

On January 13, 2026, the National Institute of Allergy and Infectious Diseases (NIAID), part of the U.S. National Institutes of Health (NIH), convened a virtual workshop to examine how advances in preventive and therapeutic HIV research can be leveraged synergistically to develop immune-based strategies to protect against HIV infection and/or disease progression. The primary objective was to review data from leading prevention trials conducted in people living without HIV and to assess how emerging strategies might be translated and evaluated in clinical studies involving people living with HIV to inform both prevention and therapeutic intervention development. Participants discussed recent immunogen design, immune monitoring, and intervention approaches relevant to both prevention and therapeutic-directed research. Here, we summarize the key findings presented at the workshop and identify scientific priorities and knowledge gaps to accelerate the development of effective immune-based strategies against HIV.

## Introduction—why now?

Despite major advances in HIV prevention and treatment, global HIV transmission remains unacceptably high and may worsen amid ongoing instability in access to care^1^. According to the U.S. Centers for Disease Control and Prevention (CDC), approximately 1.2 million people are living with HIV in the United States, with about 39,000 new infections reported annually^2^. The recent success of long-acting pre-exposure prophylaxis (PrEP) agents, including cabotegravir and lenacapavir, demonstrates that potent antiretroviral strategies can substantially reduce transmission^3,4^. However, approximately 40% of new infections occur among individuals who do not perceive themselves to be at high risk, limiting the reach of pharmacologic prevention strategies that depend on risk identification, adherence, and easy accessibility of the drug^5^. An effective HIV vaccine therefore remains essential to achieve durable, scalable, and equitable epidemic control.

Lindsey Baden, from Harvard Medical School, expanded further on this point, discussing recent advances in HIV treatment and prevention, as well as ongoing challenges in controlling the epidemic^2^. Despite progress, current interventions do not adequately reach all affected populations, and their effectiveness may decline over time due to medication fatigue, perceived changing HIV acquisition risk, and possible emergent drug resistance. In addition, although highly effective, antiretroviral-based prevention strategies can be associated with stigma^6^, underscoring the importance of individual choice and the need for diverse prevention options. Future solutions should be long-lasting, measured in years rather than months, and less dependent on sustained healthcare infrastructure.

Advances in broadly neutralizing antibody (bNAb) research, including proof-of-concept findings from the Antibody Mediated Prevention (AMP) trials, have defined key correlates of protection and identified conserved envelope (Env) epitopes susceptible to immune targeting^7,8^. Germline-targeting (GT) and sequential immunization strategies are designed to prime and mature rare B cell precursors capable of generating bNAbs with sufficient breadth and potency to prevent transmission^9^. Continued evaluation of candidate immunogens in people living with and without HIV is critical for elucidating immune priming mechanisms, guiding antibody lineage maturation, and defining the impact of pre-existing immunity on the elicitation of bNAbs. Integrating preventive and therapeutic vaccine research, including studies incorporating planned analytical treatment interruption (ATI) of antiretroviral therapy (ART)^10^, may accelerate the development of immune-based strategies that complement long-acting PrEP and ultimately contribute to both epidemic control and cure efforts.

## B cells and antibody responses

A detailed understanding of B cell biology and antibody evolution has become central to HIV vaccine, therapy and cure research. Advances in high-resolution B cell phenotyping, lineage tracing, structural biology, and functional neutralization assays allow precise evaluation of how vaccine candidates engage rare precursors, shape affinity maturation, and drive breadth. These scientific tools, combined with clinical studies in both people living with and without HIV, are accelerating progress toward rational immunogen design and clarifying how antibody responses may contribute to prevention and to durable post-treatment viral control.

**Fig. 1:**
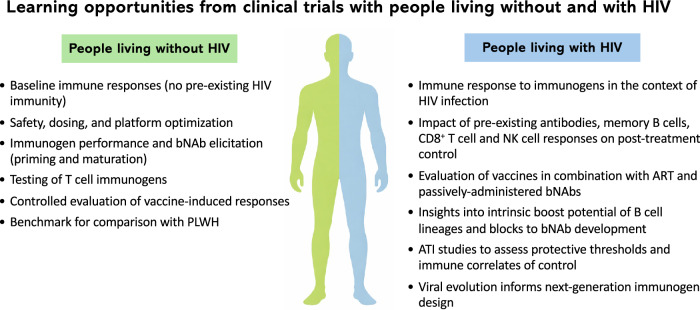
Learning opportunities from clinical trials with people living without and with HIV. Clinical trials in people living without and with HIV provide complementary insights for the development of preventive and therapeutic HIV vaccines. Studies in individuals without HIV define baseline immune responses, safety, and the capacity of vaccines to initiate broadly neutralizing antibody (bNAb) and T cell responses. In contrast, studies in people living with HIV (PLWH) assess vaccine performance in the context of pre-existing immunity, combination strategies, and viral control during analytical treatment interruption (ATI). Together, these approaches inform immunogen design and accelerate HIV vaccine development. PLWH - people living with HIV, ART - antiretroviral therapy.

Katharine Bar, from the University of Pennsylvania, discussed the bidirectional cross talk between reverse vaccinology^11^ and the development of HIV vaccines for bNAb elicitation, emphasizing that current bNAb vaccine strategies are fundamentally informed by the co-evolution of the immune response and the virus in people living with HIV (PLWH) who naturally develop breadth. She pointed out how the characterization of the immune response in PLWH and Simian–human immunodeficiency virus (SHIV)-infected animal models (see below) is guiding sequential immunization approaches, from B cell germline priming, to shepherding and boosting. The same immunization approaches are now being translated into parallel studies in PLWH to better understand the impact of baseline autologous antibody responses and immune dysfunction (Fig. 1). Bar also underscored the implications for HIV cure research, noting that B cell-targeting vaccines must be evaluated in the context of chronic infection, immune exhaustion, and combination strategies with T cell vaccines or other immunotherapies to determine whether vaccine-induced antibodies can contribute to durable, ART-free viral control.

The GT HIV vaccine strategy aims to engage naive B cells expressing germline B cell receptors with the potential to develop into bNAb lineages and to guide their maturation through sequential immunization^12^. These approaches have reliably initiated bNAb precursor lineages in preclinical models^13^ and in people without HIV^14^ (Table 1). However, early GT responses are typically assessed by B-cell activation, lineage features, and binding to engineered immunogens, and do not necessarily indicate neutralization of circulating or rebound-competent viruses. Functional neutralization generally requires further maturation through optimized boosting. Translating GT strategies to PLWH is more complex because pre-existing Env-specific immunity, heterogeneous immune histories, and diverse viral reservoirs may shape vaccine-driven responses, as noted by William Schief from Scripps Research and Moderna. GT priming, with or without boosting, may generate antibody responses that bind or weakly recognize some reservoir-derived Env variants, but such responses are unlikely to provide sufficient breadth or potency to cover all rebound-competent viruses. Moreover, because early bNAb precursors often have limited recognition of circulating or rebound viruses, their direct effect on viral rebound after ATI may be limited unless subsequent boosts drive maturation toward antibodies with broader binding and neutralizing activity. Thus, optimized boosting will be needed to generate mature bNAbs capable of recognizing and neutralizing diverse strains. In the near term, vaccine-elicited bNAbs are unlikely to replace passively infused bNAbs, given the challenges of achieving durable serum concentrations, sufficient breadth, potency, and resistance to viral escape. A complementary role is more plausible: vaccine-induced antibodies may enhance the effects of passive bNAb therapy and contribute to control rebound viruses if they acquire neutralizing activity and adequate breadth.

**Table 1:** Main ongoing or concluded prevention and/or therapeutic clinical trials in people living with and without HIV

NCT #	Title	Immunogen/Adjuvant (epitope)	Population	Approach	Main read-outs	Status/Reference
03934541	Evaluating the Safety and Immunogenicity of an HIV-1 gp41 MPER-656 Liposome Vaccine in Healthy, HIV-uninfected Adult Participants (HVTN 133)	MPER-656 Liposome Vaccine + alum (MPER)	People living without HIV (PWOH) (US)	Vaccination IM at months 0, 2 and 6 at 2 different doses	Safety and tolerability; binding Ab response to MPER-peptide specific IgG; neutralizing Ab responses	Erdmann et al., 2025^44^
06033209	A Trial to Evaluate an HIV Envelope Trimer, N332-GT5 gp140, Adjuvanted with SMNP in Adult Participants Without HIV (HVTN 144)	N332-GT5 gp140 + SMNP adjuvant (V3)	PWOH (US)	Bolus vs fractionated dosing and intramuscular vs subcutaneous dosing	Safety and tolerability; BG18-specific B cells; neutralizing antibodies	Study ongoing
05903339	Clinical Trial to Evaluate the Safety and Immunogenicity of Ferritin Nanoparticles Expressing Native-like HIV-1 Envelope Trimers Followed by Boost With mRNA Lipid Nanoparticles Encoding a Native-like HIV-1 Envelope Trimer in Adults Without HIV (HVTN 307)	V3G CH848 Pr-NP1 ferritin nanoparticle vaccine adjuvanted with 3M-052-AF + alum or ACU-026-001-1 boosted with V3G CH848 mRNA-Tr2 lipid nanoparticle (V3)	PWOH (US)	Compares 2 different adjuvants and 2 different doses of ferritin nanoparticle vaccine	Safety and tolerability; V3G-specific precursor IgM+ and IgG+ B cells; binding Abs; neutralizing Ab responses; CD4^+^ T-cell responses;	Study ongoing
07390474	Clinical Trial to Evaluate the Safety and Immunogenicity of the V2 Apex-Directed Immunogens DV201P-RNA and DV202B1-RNA in Adult Participants Without HIV (HVTN 322)	DV201P-RNA boosted with DV202B1-RNA vaccine (V2)	PWOH (US)	Compares 3 different doses of each RNA vaccine	Safety and tolerability; V2 apex specific B cells; neutralizing Ab responses; binding Abs;	Study ongoing
04915768	Evaluating the Safety and Immunogenicity of Stabilized CH505 TF chTrimer in Healthy, HIV-uninfected Adult Participants (HVTN 300)	CH505 TF chTrimer vaccine with 3M-052-AF +/- Alum adjuvant (CD4-bs, V2, V3)	PWOH (US)	Vaccination with immunogen 5x in Part A; varying amounts of protein and adjuvant in Part B	Safety and tolerability; CD4-bs B cells; V2 and V3 B cells; CH505TF B cells; neutralization of tier 2 strains;	Walsh et al., 2026^45^
04224701	A Clinical Trial to Evaluate the Safety and Immunogenicity of Recombinant HIV-1 Envelope Protein BG505 SOSIP.GT1.1 Gp140 Vaccine, Adjuvanted in Healthy, HIV-uninfected Adults (C101)	BG505 SOSIP.GT1.1 gp140 trimer+ AS01_B_ adjuvant (CD4-bs)	PWOH (US, Netherlands)	High and low dose of the vaccine compared to placebo	Safety and tolerability; Frequency and magnitude of binding antibody responses to GT1.1 trimer	Caniels et al., 2025^27^
06006546	The Study of Immunization in People Living With HIV Undergoing an ATI for Elicitation of VRC01-lineage Antibodies (HVTN 807)	426c.Mod.Core-C4b adjuvanted with 3M-052-AF + alum (CD4-bs)	PLWH (US)	Vaccination followed by ATI	Safety and tolerability; Env and CD4-bs-specific B cells; VRC01 class BCR sequences; neutralizing activity; viral sequences between arms	Study ongoing
N/A	RENEW trials: monitoring antibody responses re-stimulated by BG505 GT1.1 immunization in PLWH under ART^a^	BG505 GT1.1 adjuvanted with 3M-052-AF + alum (CD4-bs)	PLWH (Switzerland and South Africa)	Vaccination followed by ATI	Antibody titers; antibody breadth; bNAb lineage reboosting	Study ongoing
04319367	A Randomised Placebo Controlled Trial of ART Plus Dual Long-acting HIV-specific Broadly Neutralising Antibodies (RIO)	3BNC117-LS and 10-1074-LS bNAbs (CD4-bs, V3 loop)	PLWH (UK)	Administration of bNAbs followed by ATI	Time to viral rebound within 20 weeks after initial ATI between bNAb group and placebo group	Fumagalli et al. 2025^24^
06680479	Safety and Immunogenicity of Stabilized CH505 TF chTrimer Vaccination in Adults Living With HIV-1 on Suppressive Antiretroviral Therapy (ACTG A5422)	CH505 TF chTrimer vaccine with 3M-052-AF +/- Alum adjuvant (epitope agnostic)	PLWH (US)	Vaccination followed by ATI	Study-related adverse events; heterologous neutralizing responses between arms	Study ongoing
03041012	Early Administration of Romidepsin and 3BNC117 in Treatment-naive HIV Patients Starting ART (eCLEAR)	Romidepsin (latency reversing agent) and/or bNAb 3BNC117 (CD4-bs)	PLWH (Denmark, UK)	Treatment followed by ATI	Safety and tolerability; Plasma HIV RNA kinetics; Quantification of the size of the proviral HIV reservoir; Time to viral rebound during ATI	Gunst et al. 2022^46^
02919306	A trial of a gorilla adenovirus vectored networked epitopes vaccine, administered to healthy adults living without and with HIV (IAVI C114)	GRAdHIVNE1 vaccine (CD8-networked epitopes)	PWOH, PLWH (Zimbabwe, South Africa)	Vaccination versus placebo	Safety and cellular response	Study ongoing
02919306	Safety and efficacy study of vaccine schedule with Ad26.Mos.HIV and MVA-Mosaic in human immunodeficiency virus (HIV)-infected adults (RV405)	Ad26.Mos.HIV MVA-Mosaic (epitope agnostic)	PLWH (Thailand)	Treatment followed by ATI	Safety, duration of viremic control, cellular and humoral immune responses	Corby et al., 2020^47^
06484335	Approach to control HIV with immune enhancement and vaccination (ACHIEV) (RV630)	VRC07-523LS and PGDM1400LS mAbs, plus ChAdOx1.tHIVconv1, ChAdOx1.HIVconsv62, MVA.tHIVconsv4 and A244d11gp120/ALFQ immunogens/adjuvant (multiple bNAb epitopes, T cell epitopes)	PLWH (Thailand)	Administration of mAbs +/-vaccination followed by ATI	Safety of mAbs in combination with vaccinations; viral load setpoint during ATI	Study ongoing

*MPER* membrane-proximal external region, *N/A* non-available, *CD4-bs* CD4-binding site, *ATI* Analytical Treatment Interruption, *ALFQ* Army Liposome Formulation containing the saponin QS-21 (Quillaja Saponaria-21), *ART* anti-retroviral therapy.

^a^Focus on participants who produce bNAbs (“bNAbers”) and non-bNAbers.

Barton Haynes, from Duke University, highlighted the HVTN 300 (NCT04915768) discovery medicine clinical trial, which evaluated the CH505 Trimer administered with 3M-052-AF/Alum for expansion of CH103-like B cell precursors, which target the CD4-bs^15^. He also described HVTN 307 clinical trial (NCT05903339; Table 1), which is comprised of a sequential immunization strategy with protein nanoparticles and mRNA boosts that successfully primed and recalled V3-glycan-specific N332-dependent B cell clones in humans. The results provide proof-of-principle that defined bNAb lineages can be initiated and longitudinally expanded through rational boosting. The next frontier for expediting the development of new vaccine candidates comes from immunologic studies in PLWH. bNAb-eliciting vaccines may achieve enhanced maturation and functional durability in PLWH, whose immune system is characterized by expanded T follicular helper cells, reduced regulatory constraints, and B cell repertoire features favoring long CDR3 and polyreactivity, as previously reported by Haynes and colleagues^16,17^. Collectively, these findings establish clinical proof-of-principle for lineage-based vaccine design and provide a mechanistic roadmap toward both preventive and therapeutic bNAb-inducing strategies.

While vaccination can elicit bNAbs, it is also fundamental to understand the role of autologous neutralizing antibodies (aNAbs), which are frequently observed in PLWH. Here, aNAbs refer to antibodies that neutralize an individual’s own HIV variants, rather than diverse heterologous strains. aNAbs, though often overlooked due to their strain specificity, can exert potent control over subsets of reservoir viruses and significantly influence time to viral rebound after ART interruption. Robert Siliciano, from Johns Hopkins University, explained that among reservoir viruses, those resistant to existing aNAbs are more likely to drive rebound and that aNAbs can suppress sensitive reservoir viruses with inhibitory potential, correlating strongly with delayed rebound in ATI studies^18^. In a striking clinical example, durable ART-free remission was associated with high inhibitory potential of polyclonal aNAbs, reaching levels comparable to ART. Notably, monoclonal aNAbs isolated from a person living with HIV can have, on an equimolar basis, inhibitory potential equal to or greater than some bNAbs (e.g., 10-1074, VRC01 or 3BNC117). This greater inhibitory potential can be measured in vitro by the steepness of the curve of inhibition versus antibody concentration, i.e., Hill coefficient^19,20^. In fact, Siliciano proposed that the Hill coefficient might more truthfully capture the cooperative nature of the epitope-antibody interaction compared to inhibitory dilution titer (PT_80_), which is extrapolated from a single point on the assay curve^21^. These findings suggest that aNAbs not only shape viral evolution but may offer important insights for both therapeutic and preventive vaccine strategies.

Marina Caskey, from Rockefeller University, reviewed recent advances in the therapeutic delivery of bNAbs for HIV, highlighting how insights from natural antibody-virus co-evolution have informed passive immunotherapy strategies. Multiple clinical trials now demonstrate that potent, long-acting bNAbs, particularly in combination with long-acting ART^22^, can maintain viral suppression in over 90% of PLWH, with dosing intervals ranging from monthly to every six months. In cure-focused studies involving ATI, a subset of participants, approximately 30% across several trials, exhibited sustained virologic control even after the concentration of antibodies in plasma decayed, a phenomenon termed the post-bNAb effect. Emerging data suggest that this control is associated with baseline host immunity, including aNAb activity, robust CD8^+^ T cell proliferative capacity^23^, and possibly NK cell function, which may be enhanced or triggered by the administration of bNAbs^24^. Reservoir analyses showed modest declines in intact proviruses among early ART-treated PLWH, though immunologic mechanisms appear more strongly linked to durable post-intervention control rather than reservoir size alone. Caskey also discussed alternative delivery approaches, such as adeno-associated virus (AAV)-mediated bNAb expression, where preclinical results in non-human primates (NHP) suggest that early-life administration may improve durability and reduce anti-drug antibody responses^25^, underscoring both the promise and ongoing challenges of antibody-based HIV therapies.

## Characterization of the immune response of PLWH to vaccine and/or ATI

PLWH are uniquely Env-experienced, with diverse, pre-existing Env-reactive antibodies and memory B cells shaped by prolonged antigen exposure and viral evolution. Penny Moore, from the University of the Witwatersrand, showed her teams’ efforts to investigate that concept in well-characterized cohorts (e.g., CAPRISA and the Swiss HIV Cohort Study^24^) and trials such as RENEW^26^. She highlighted the comparison of participants who develop bNAbs versus participants who do not to investigate whether a germline-targeting Env trimer (e.g., BG505.GT1.1^27^) could stimulate on-target lineages, increase bNAb titers, and/or move non-bNAb responders toward breadth while on ART. Moore also emphasized that these approaches can reveal parameters associated with protection, the capacity of B cell lineages to be triggered or boosted, and the immunologic blocks to breadth development across different geographies and host genetics. However, she noted that the added scientific value of post-vaccination ATI must be weighed carefully and follow strict criteria to assure, first and foremost, participants’ safety^10^.

PLWH with chronic HIV exhibit a diverse Env-specific memory B cell response, including partially-matured CD4-bs bNAb precursors, that may be further activated and matured by vaccination and antigen exposure during ATI. Leonidas Stamatatos, from the Fred Hutchinson Cancer Center, talked about HVTN 807 (NCT06006546), a proof-of-principle Phase I clinical trial evaluating the germline-targeting 426 c.Mod.Core-C4b self-assembling nanoparticle in PLWH on long-term ART, which is the same immunogen successfully evaluated in people living without HIV in HVTN 301 (NCT06796686). The preliminary results from the trial show that, despite substantial pre-existing anti-Env and anti-426c.Mod.Core antibodies, including variable levels of circulating CD4-bs-directed IgG, vaccination increased the frequency of CD4-bs-specific B cell responses, indicating that 426c.Mod.Core can expand target precursor populations even in the context of baseline immunity. Ongoing analyses of emerging viral envelopes aim to determine whether rebound variants possess features capable of further maturing vaccine-elicited B cell lineages toward broader neutralization. Collectively, these findings support the feasibility of CD4-bs germline-targeting vaccination in PLWH and provide early insights into virus–antibody dynamics during treatment interruption.

Asier Saez-Cirion, from the Pasteur Institute, presented new insights into mechanisms underlying post-ART control of HIV, drawing primarily from the Viro-Immunological Sustained Control after Treatment Interruption (VISCONTI) cohort^28^. Among 32 individuals who maintained long-term viral control after ART interruption, 19 were “silent” controllers (i.e., without detectable viremia), while 13 were “active” controllers (i.e., re-established control after low viremia peaks). A key finding was the association of a distinct immunogenetic fingerprint, characterized by overrepresentation of HLA-B*35 in controllers, which is an allele paradoxically associated with rapid progression of disease in the absence of ART. The influence of HLA-B*35 was observed in combination with HLA class I alleles expressing Bw4 and C2 ligands of killer immunoglobulin-like receptors (KIRs), which are combinations linked to NK cell education. Interestingly, prospective clinical data further demonstrated that higher baseline NKG2A^+^ NK cell levels predicted better outcomes following ATI. Complementary analyses revealed that post-treatment controllers also develop coordinated immune responses, including polyfunctional antibodies and CD8^+^ T cells with stem-like properties (TCF-1^high^, PD-1^low^) that acquire potent antiviral activity after ART interruption, in contrast to non-controllers^29^. Notably, similar associations between NKG2A^+^ NK cell frequencies and post-treatment control were observed in a NHP model of early-treated SIV infection^30^. Collectively, these data support a model in which calibrated innate and adaptive immune programs, rather than classical hyperactivated effector responses, promote sustained post-ART viral control.

Reviewing strategic considerations for therapeutically targeting HIV-specific CD8⁺ T cell responses, Rachel Rutishauser, from the University of California, San Francisco, emphasized that effective post-treatment control will require responses that are potent, durable, broadly reactive against autologous and evolving viruses, and possibly localized to tissue sites of viral persistence. Therapeutic vaccination might elicit such responses, but a central unmet need is induction of stem-like CD8⁺ T cells^31^ with high proliferative capacity and long-lived antiviral potential, as mentioned above. She highlighted that different vaccine platforms, such as nucleic acid (DNA and mRNA), viral vectors, proteins (trimers and nanoparticles), as well as the use of adjuvants, can help shape the desired immune responses. Rutishauser stressed that correlates of control often emerge during the early rebound window, characterized by rapid clonotypic expansions of effector CD8⁺ T cells, which can be investigated by sensitive assays involving tetramers and TCR sequencing rather than by standard stimulation assays^32–34^. Rational antigen selection, attention to T cell quality (stemness and effector functions), and standardized, sensitive assays are critical priorities for advancing therapeutic CD8⁺ T cell vaccines^35^.

## Novel approaches and tools

Rapid progress in HIV vaccine research has been driven by new experimental models, advanced immunologic profiling, and artificial intelligence (AI), enabling deeper analysis of B and T cell responses, and viral dynamics. These tools are critical for understanding how candidate immunogens engage rare B cell precursors, guide bNAb maturation, and shape virus–antibody co-evolution in both preventive and therapeutic settings.

SHIVs, chimeric viruses combining HIV-1 Env with an SIV backbone, enable in vivo investigation of HIV Env immunobiology in outbred Rhesus macaques. Enhanced Env-CD4 binding affinity permits sustained replication without altering native antigenicity, neutralization sensitivity, or conformational integrity, making SHIV a robust model of Env–antibody co-evolution^36^. Notably, SHIV infection recapitulates the tempo and frequency of bNAb development in humans, underscoring its translational relevance while highlighting the need for optimization to achieve faster, more consistent bNAb induction^37,38^. Beatrice Hahn, from the University of Pennsylvania, described two SHIV models that markedly enhanced the efficiency and speed of bNAb elicitation in Rhesus macaques. In the SHIV.5MUT model, based on a BG505 Env with four V1 loop mutations, 64% of animals developed V3-glycan bNAbs with heterologous breadth within one year, unlike the parental virus. These early V1-directed autologous responses drove rapid viral escape, exposing the V3 supersite and priming diverse bNAb precursors. The shared escape mutations preceding breadth helped inform rational boost design^39^. In a second model, SHIV.OPT4 and SHIV.OPT4-shielded elicited V2 apex bNAbs in nearly all macaques within a year, with up to 90% breadth. Those Envs enhanced precursor priming by 40- to 370-fold, and co-evolution analyses identified key mutations linked to breadth. Now, aPhase I clinical trial is evaluating the safety and immunogenicity of V2 apex-directed immunogens in people without HIV (NCT07390474). Together, these models provide a mechanistic blueprint for epitope-focused priming and evolution-guided boosting to accelerate bNAb maturation.

Immunogens can be rationally designed to select for specific antibody mutations^40^, however, to accelerate antigen optimization and reduce reliance on sequential boosting, large-scale antibody variant datasets are increasingly integrated into artificial intelligence (AI)-guided antigen selection platforms. These approaches enable the identification of antigen features that drive selection of desired antibody responses and allow rapid in silico screening of large antigen libraries. At the Duke Center for HIV/AIDS Vaccine Development (CHAVD), led by Barton Haynes, Project 2K (<https://www.managedhealthcareexecutive.com/view/ai-set-to-speed-up-hiv-vaccine-research-and-trials-focusing-on-fair-access>) leverages AI to refine immunogen design by analyzing binding breadth, affinity, and thermal stability across 2000 antibody fragment variants representing diverse clonal lineages targeting multiple Env epitopes. This dataset is being further expanded with antibodies isolated from clinical trials, including HVTN 300 and HVTN 307.

David Montefiori, from Duke University, described the in vitro assessment of the immune response to HIV antigens tested in clinical trials using the TZM-bl pseudovirus neutralization assay^41^, in which antibody-mediated reductions in luminescence quantify the inhibition of viral entry. Using engineered precursor detection pseudoviruses, designed with features that enhance binding to germline-targeting unmutated common ancestors, early bNAb lineage activation targeting the CD4-bs, V3 glycan, or V2 apex can be quantified. On-target activity is confirmed using matched epitope knockout viruses, where a ≥3-fold reduction in neutralization indicates a lineage-specific signature. Across multiple clinical studies, detection of these precursor signatures has been validated by monoclonal antibody isolation and structural analyses, providing confidence that measured responses reflect authentic bNAb lineages. Evidence of maturation is demonstrated by neutralization assays performed with progressively less modified viruses and ultimately heterologous tier 2 virus panels, indicating increasing breadth and potency. In GT bNAb vaccination trials in PLWH, additional markers of success include improved heterologous neutralization magnitude and breadth, expanded neutralization of autologous reservoir viruses, rapid neutralization of autologous rebound viruses, monitoring of escape variants, and emergence or maturation of bNAb precursor signatures absent at baseline, linking immunization to functional antiviral activity.

Accurate quantification of antibody neutralizing activity in plasma from PLWH on ART, including long-acting agents such as lenacapavir, is essential for evaluating therapeutic vaccines. Alexandra Trkola, from the University of Zurich, presented ART-DEX, a size-exclusion–based assay that exploits the small molecular size and pH-dependent plasma protein binding of ART drugs, incorporating sequential alkaline and acidic dissociation steps followed by size-exclusion filtration to remove ART while preserving antibody function, thereby permitting reliable TZM-bl neutralization assays^42^. To further eliminate the effects of ART, the method has been combined with HIV pseudoviruses harboring a multidrug-resistant backbone and further optimized into a double ART-DEX protocol with repeated filtration steps, reducing nonspecific inhibition to near-undetectable levels. The assay has been validated using clinically relevant drug concentrations and diverse plasma samples, demonstrating preserved neutralization potency despite chemical treatment. Now widely implemented in bNAb durability and interventional trials, ART-DEX represents a robust, high-throughput alternative to antibody purification, with ongoing quality control adjustments (e.g., adoption of 7 kDa cutoff filtration plates) to ensure assay reliability.

Rachael Parks, from the Fred Hutchinson Cancer Center, described integrated B cell analysis pipelines used in HVTN preventive and ATI clinical trials to evaluate whether germline-targeting immunogens successfully initiate and mature bNAb lineages. Flow cytometry is used to analyze peripheral blood mononuclear cells from vaccine recipients to define B cell populations and characterize antigen- and epitope-specific memory B cells. This is achieved by staining cells with fluorescently labeled, vaccine-matched Env probes together with matched epitope-knockout probes (lacking the epitope of interest) in multiparametric flow-based assays. These approaches enable both quantification and sorting of vaccine-specific B cells for downstream analyses. Sorted epitope-specific B cells are then subjected to single-cell B cell receptor sequencing by 10x Genomics^TM^, followed by downstream bioinformatic processing to generate high-quality paired heavy- and light-chain sequences for interrogation of bNAb-class genetic features, such as V gene usage and CDR characteristics^43^. This approach has been applied across multiple prevention trials, including G001, G002, G003, HVTN 300, HVTN 301, and HVTN 305 investigating CD4-bs immunogens, as well as HVTN 144 and HVTN 307 targeting the V3 glycan epitope, to confirm activation of target bNAb precursor lineages. In studies involving PLWH (e.g., HVTN 807), Parks emphasized that pre-existing antibodies, memory B cells, and altered B cell phenotypes might necessitate methodological adaptations. Specifically, baseline measurements are essential to distinguish true vaccine-induced responses from pre-existing immunity, and expanded flow cytometry panels might be required to characterize activated, exhausted, or atypical B cell subsets, in the context of HIV infection. Additionally, probe design may need to be refined to detect more mature or partially evolved bNAb precursors that are already present in some PLWH. Collectively, these adaptations are critical to accurately interpret B cell responses and lineage progression in the distinct immunologic context of HIV infection.

## Bridging preventive and therapeutic HIV vaccine development

The workshop highlighted the increasing convergence of preventive and therapeutic HIV vaccine research, demonstrating that advances in immunogen design, immune monitoring, and clinical trial methodologies are creating new opportunities to accelerate progress toward both durable protection and improved therapies. Emerging experimental approaches, including optimized SHIV models together with advanced immunologic assays, such as high-resolution B cell receptor sequencing, single-cell profiling, and sensitive neutralization assays, are enabling deeper characterization of vaccine-elicited immune responses and virus–host interactions.

However, there is a need to more effectively integrate discoveries from preventive and therapeutic HIV vaccine research. Immune responses observed in PLWH can provide unique insights into protective mechanisms that are difficult to identify in HIV-uninfected populations. For example, characterization of aNAb responses, naturally occurring bNAb lineages, and post-treatment control phenotypes may reveal new immune pathways, host factors, and parameters that can be leveraged to design next-generation preventive vaccines. Conversely, advances in GT immunogens, sequential immunization strategies, and immune monitoring platforms developed in prevention trials offer powerful tools to interrogate and enhance immune responses in PLWH. A major knowledge gap therefore lies in understanding how immune responses generated, maintained, and shaped during chronic infection can be translated into vaccine strategies that induce comparable responses in people without HIV, while also identifying approaches to boost or redirect these responses toward durable ART-free viral control in therapeutic settings. Addressing this challenge will require harmonized studies across populations, standardized immunologic endpoints, and integrated analyses of host and viral parameters.

Despite many advances, several additional gaps remain. First, the mechanisms governing elicitation and maturation of vaccine-induced bNAb lineages in the context of pre-existing immunity remain incompletely understood. Second, correlates of durable viral control following therapeutic interventions need to be further defined, particularly with respect to the interplay between antibodies, CD8⁺ T cells, NK cells, and viral reservoir dynamics. Third, standardized, sensitive assays and harmonized endpoints are needed to enable comparisons across preventive and therapeutic studies, especially those incorporating ATI. Finally, improved computational and AI-based approaches are needed to accelerate vaccine development and immune response analysis. AI-driven platforms have the potential to inform immunogen design by leveraging large datasets generated from clinical trials, including bNAb characterization and lineage analyses. Similarly, more robust and standardized computational pipelines are needed to integrate, harmonize, and expedite the analysis of increasingly complex B cell receptor sequencing and other high-dimensional immunologic datasets. To address these gaps, it is critical to evaluate vaccine candidates in people living with and without HIV and to leverage ATI studies to better understand mechanisms contributing to immune control of HIV.
